# Systematic Characteristic Exploration of the Chimeras Generated in Multiple Displacement Amplification through Next Generation Sequencing Data Reanalysis

**DOI:** 10.1371/journal.pone.0139857

**Published:** 2015-10-06

**Authors:** Jing Tu, Jing Guo, Junji Li, Shen Gao, Bei Yao, Zuhong Lu

**Affiliations:** State Key Lab of Bioelectronics, School of Biological Science and Medical Engineering, Southeast University, Nanjing, China; The University of Hong Kong, HONG KONG

## Abstract

**Background:**

The chimeric sequences produced by phi29 DNA polymerase, which are named as chimeras, influence the performance of the multiple displacement amplification (MDA) and also increase the difficulty of sequence data process. Despite several articles have reported the existence of chimeric sequence, there was only one research focusing on the structure and generation mechanism of chimeras, and it was merely based on hundreds of chimeras found in the sequence data of E. *coli* genome.

**Method:**

We finished data mining towards a series of Next Generation Sequencing (NGS) reads which were used for whole genome haplotype assembling in a primary study. We established a bioinformatics pipeline based on subsection alignment strategy to discover all the chimeras inside and achieve their structural visualization. Then, we artificially defined two statistical indexes (the chimeric distance and the overlap length), and their regular abundance distribution helped illustrate of the structural characteristics of the chimeras. Finally we analyzed the relationship between the chimera type and the average insertion size, so that illustrate a method to decrease the proportion of wasted data in the procedure of DNA library construction.

**Results/Conclusion:**

131.4 Gb pair-end (PE) sequence data was reanalyzed for the chimeras. Totally, 40,259,438 read pairs (6.19%) with chimerism were discovered among 650,430,811 read pairs. The chimeric sequences are consisted of two or more parts which locate inconsecutively but adjacently on the chromosome. The chimeric distance between the locations of adjacent parts on the chromosome followed an approximate bimodal distribution ranging from 0 to over 5,000 nt, whose peak was at about 250 to 300 nt. The overlap length of adjacent parts followed an approximate Poisson distribution and revealed a peak at 6 nt. Moreover, unmapped chimeras, which were classified as the wasted data, could be reduced by properly increasing the length of the insertion segment size through a linear correlation analysis.

**Significance:**

This study exhibited the profile of the phi29MDA chimeras by tens of millions of chimeric sequences, and helped understand the amplification mechanism of the phi29 DNA polymerase. Our work also illustrated the importance of NGS data reanalysis, not only for the improvement of data utilization efficiency, but also for more potential genomic information.

## Background

The whole genome amplification (WGA) methodologies have been developed for the purpose of human genome researches recently[1]. Initial WGA approaches were mainly based on the degenerate oligonucleotide primed polymerase chain reaction (DOP-PCR), which could amplify the product DNA several orders of magnitude than the initial template DNA [2, 3]. However, these approaches could lead to the nonspecific amplification and the amplification bias. Recent reports have described the use of non-PCR-based linear amplification protocols for WGA, and the bacteriophage phi29 DNA polymerase has been applied for the efficient amplification of circular DNA viral genomes without the need of specific primers in DOP-PCR [4, 5]. The phi29 DNA polymerase mediates an isothermal multiple displacement amplification (MDA) process, which omits the step to use the PCR instrument for the limiting initial DNA [6, 7]. It has high processivity and proofreading activity, and can generate large fragments (≥10K bp) by using small template size (≥10 ng) with a good coverage rate and a reduced amplification bias [8]. Due to the obvious advantages, the phi29 DNA polymerase is being widely used in the procedure of microscale initial genomic DNA (gDNA) amplification in the WGA [9].

The chimeric sequence reads (*i*.*e*. chimeras) are consisted of two or more parts which locate inconsecutively but adjacently on the chromosome. In 2006, Zhang, K. *et al*. found chimeras in the whole genome shotgun sequence data of single-cell *Prochlorococcus* for genomic assembling, but the generation and structural details of chimerism lacked of enough elaboration [10]. Roger S Laken *et al*. used the 454 sequence platform to discover the phi29MDA chimeras in the sequence data of E. *coli* in 2007 [11]. However, this primary study was based on low-throughput sequence platform, and its analysis was not so explicit to explain the mechanism of phi29MDA chimera generation. Hundreds of chimeras (475 chimeras) was fairly far away from the significant level, which led to the lack of representativeness. Meanwhile, the genomic simplicity of the E. *coli* could not equivalently reflect the chimerism happening in human genome sequence data. With the increasingly usage of MDA in NGS, the phi29MDA chimerism could lead to severe disturbance in many studies, especially those focusing on heterogeneity and haplotype. Therefore, it is of great importance to elaborate the phi29MDA chimerism in WGA. From this standpoint, chimerism in human genome NGS data on the high-throughput sequence platform should be systematically studied.

Herein, we made further analysis focusing on phi29MDA human genome sequence data with the size over 130 Gb, which was used for whole genome haplotype assembling in 2013 [12]. For the purpose to systematically illustrate the regular patterns of the chimerism, we paid close attention to all the non-pair-end alignment reads which were easier to be abandoned in conventional bioinformatics pipeline. Through our reanalysis to hundreds of millions of PE sequence reads, the results revealed the diversification of varied-level chimeras, and the statistically significant proportion of total chimeras in two samples (6.37% and 5.93% respectively, the detailed numbers were 24595400 and 15664038 PE read pairs respectively). Our study exhibited the profile of the phi29MDA chimerism through tens of millions of chimeras, and helped understand the amplification mechanism of the phi29 DNA polymerase.

## Results

### Data downloading and initial processing

The initial 206.8 Gb sequence data was downloaded from the Sequence Read Archive (SRA) database of National Center for Biotechnology Information (NCBI). According to the ethnic groups, the data was separated into two samples (SRX247249 and SRX252522). Since the existence of reads with N would influence the normalization of the following reanalysis, we filtered those reads with N, and 131.4 Gb raw reads was obtained after this procedure. The pair-end (PE) raw reads of each sample were aligned by Short Oligonucleotide Alignment Program 2 (SOAP2) in the PE alignment mode[13]. The data production was demonstrated as Table 1.

**Table 1:** General data production.

**Total Reads**	**Sample ID**	**Mapped**	**Unmapped**
772587692(386293846 pairs)	**SRX247249(101bp)**	726523674(94.04%)	46064018(5.96%)
	**SRX247249(30bp)**	757652247(98.07%)	14935445(1.93%)
528273930(264136965 pairs)	**SRX252522(101bp)**	498760707(94.41%)	29513223(5.59%)
	**SRX252522(30bp)**	518496783(98.15%)	9777147(1.85%)

### Definition and visualization of the phi29MDA chimeras

**Fig 1:**
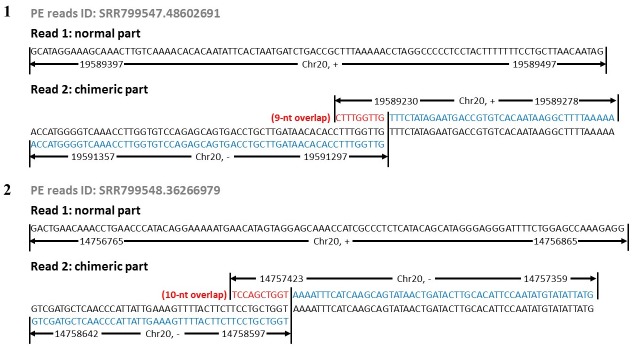
The visualization of the chimeras with the read parts located on the reverse strands (1) or on the same strands (2).

The chimeras are consisted of two or more parts which locate inconsecutively but adjacently on the chromosome. Differing from the MDA primer polymer which are consisted of two or more sequence primers with the concordant length of 20–25 bp, the phi29MDA chimeras had at least one chimeric part whose length was definitely no shorter than 30 bp based on our bioinformatics pipeline (Materials and Methods), which meant the primer polymers would not mistakenly classified as the chimeras in our bioinformatics pipeline. On the aspect of the internal structure among the chimeric parts, there were overlap segments between the tail of the former part and the head of the following part. As for the chimeric strand specificity, this phenomena not only happened between two reverse strands, but also was observed on the same strand. Following the nomenclature of the previous research [11], fundamental chimerism could be divided into two types: inverted chimeras and direct chimeras. Two subsections of inverted chimeras are aligned to two reverse strands, while two subsections of direct chimera could be aligned concordantly to one strand. (Fig 1).

### Proportion statistics of total and varied-level chimeras

**Fig 2:**
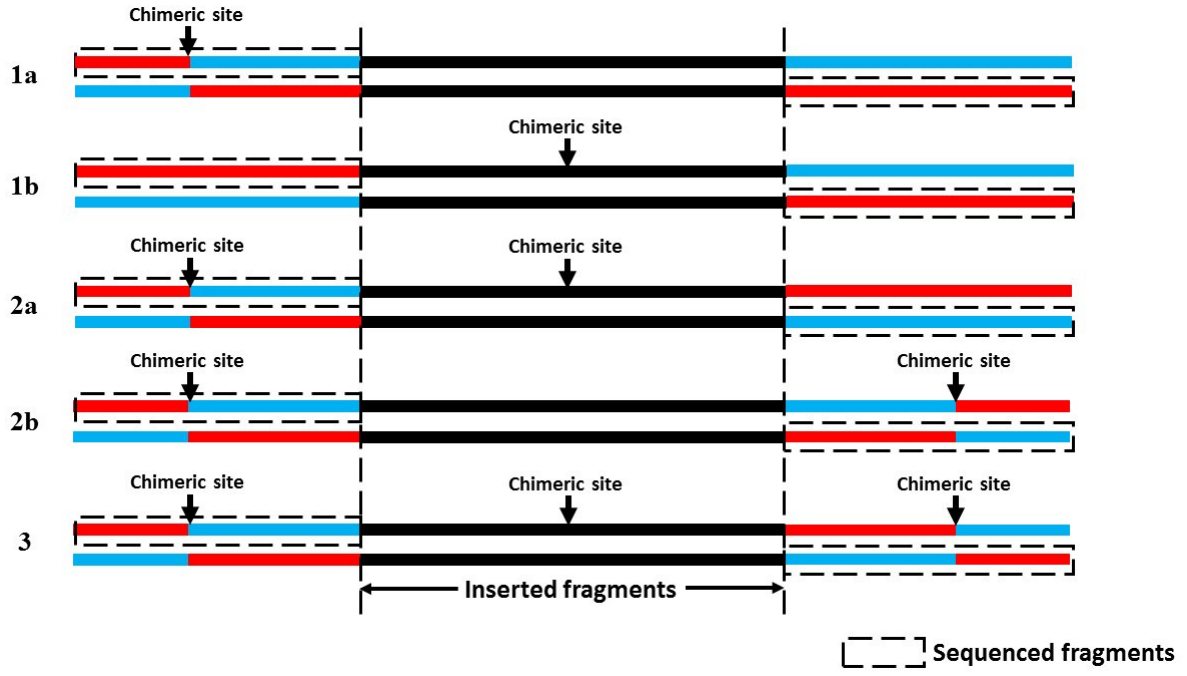
The characteristics of the sequence data of the varied-level chimeras. (1a) (1b) 1-level chimera; (2a) (2b) 2-level chimera; (3) 3-level chimera.

Since the segment chimerism may happen for more than one times on a PE chimera read pair, it was necessary to classify the chimeras according to the levels of chimerism. Therefore, we generally separated the chimeras into three categories (1-level, 2-level and 3-level). For a 1-level chimera, only one time of chimerism happened, which lead to two sub-categories: the insertion chimera (the chimerism happened in the insertion segments) and the pair-end chimera (the chimerism happened in the pair-end sequence reads). In similar ways, the 2-level and 3-level chimeras could be classified into several sub-categories. (Fig 2).

Totally, 24595400 (6.37%) and 15664038 (5.93%) PE read pairs were classified as chimeras in sample SRX247249 and SRX252522 respectively, which were both statistically significant. Moreover, the counting of the varied-level chimeras was finished according to the artificial classification of the chimeras above (Table 2). The results revealed that the majority of the chimeras (97.06% for SRX247249 and 95.71% for SRX252522 respectively) was in 1-level, and the higher-level chimeras were two orders of magnitude less than 1-level chimeras.

**Table 2:** The amount of varied-level chimeras and the total proportion of chimeras.

**Sample ID**	**Total read pairs**	**1-level chimeras**	**2-level chimeras**	**3-level chimeras**	**Total proportion**
**SRX247249**	386293846	23872631	564397	158372	6.37%
**SRX252522**	264136965	14992729	445861	225448	5.93%

**Fig 3:**
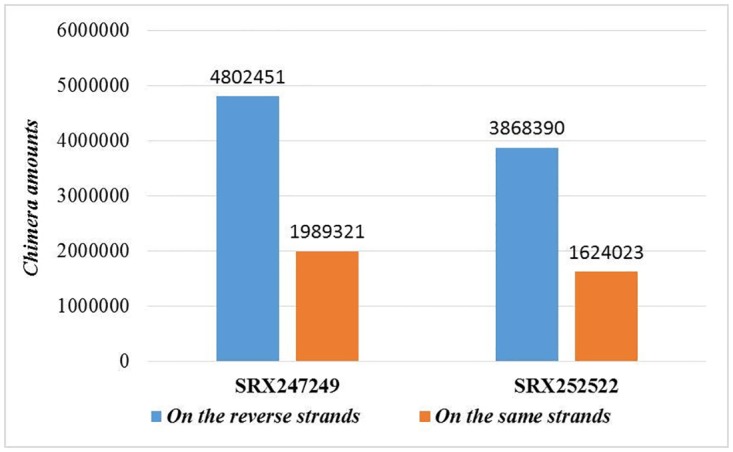
The quantity statistics of the pair-end chimeras with two parts on the reverse strands or on the same strands.

Since the segments of the chimeras could locate on either the same strands or the reverse strands, we also finished the quantity statistics of the pair-end chimeras, whose chimeric characteristics were visible, to see the related proportion of these two types (Fig 3). The results illustrated that the chimeric action could be easier to happen between two reverse strands (70.71% and 70.43% for each sample), which was generally in concordance with the results of previous researches [11].

Furthermore, according to our statistics, 74.55% (76.51% for SRX247249 and 71.47% for SRX252522 respectively, *i*.*e*. 4.61% of the total read pairs) of chimerism caused by phi29MDA in this series of sequence data happened in the insertion segments of pair-end reads. It led to the proportion increasement of the single-end alignment. However, the single-end mapped data is available for most ordinary bioinformatics researches. The remaining 25.45% chimeric read pairs (1.58% of the total read pairs) were the traditionally defined chimeras, which had at least one end with directly visual chimerism.

### Distribution of the chimeric distance and the overlap length in 1-level chimeras

**Fig 4:**
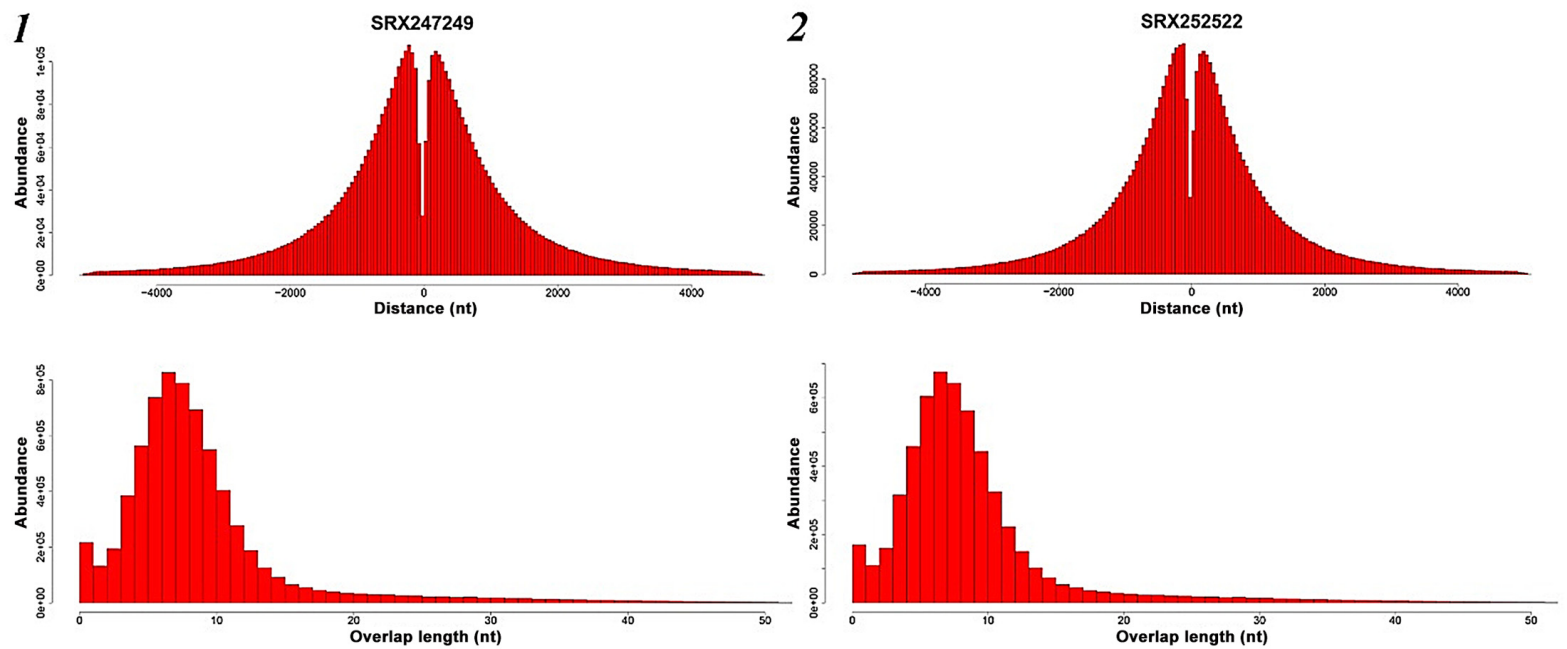
The abundance distribution graph of the physical genomic distance of the two parts of the reads and the length of the overlap segments. Sample SRX247249 was illustrated in Fig 4–1, and Sample SRX252522 was illustrated in Fig 4–2.

Theoretically, according to the enzyme kinetics of the phi29 DNA polymerase [14–16], as long as the overlap segments exists between two chimeric parts, meanwhile the chimeric distance was not very long, there was the probability to generate the chimeras. To assess the characteristics of the chimeras, we chose the chimeras with two chimeric parts on the reverse strands as the candidates, which had the largest number and the simplest structure. We analyzed the chimeric distance and the overlap length, and gave out the abundance distribution graph of the two samples (Fig 4). The results revealed that the abundance of the chimeric distance revealed an approximate bimodal distribution ranging from 0 to 5,000 nt, whose peak values was approximately 250 to 300 nt, *i*.*e*. the most probable chimeric distance. Furthermore, the abundance of the overlap length was observed as an approximate Poisson distribution with a peak of 6 nt, in which most chimeras had overlap segments ranging from 5 to 8 nt.

### Relationship between the average insertion size and the chimera type

**Fig 5:**
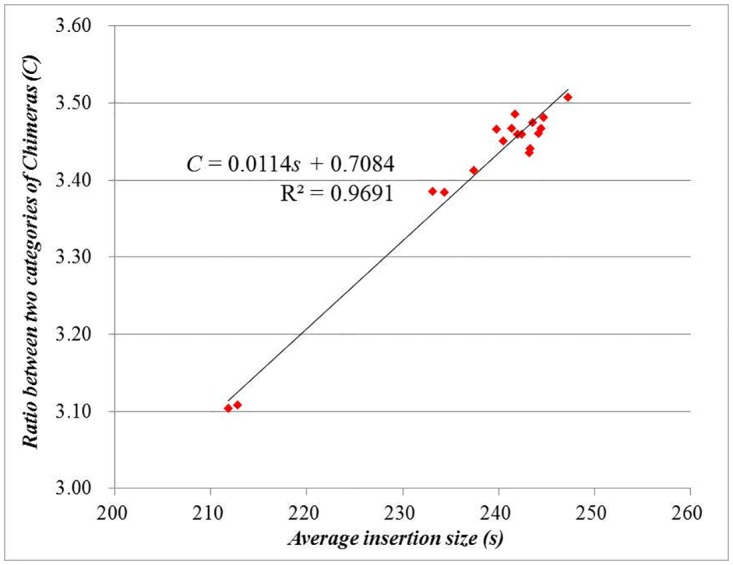
The curve of the 18 subsamples about the relationship between the average insertion size and the ratio of the insertion chimeras to the pair-end chimeras.

All the chimeras we obtained could be alternatively separated into two kinds: insertion chimeras and pair-end chimeras. Under normal circumstances, insertion chimeras could be regarded as available data because their reads are completely mapped to the reference. However, the pair-end chimeras tend to be classified into the wasted data due to their inconformity with the genome. As the production of chimeras could not be avoided by using phi29MDA, it is necessary to investigate which factor directly influences the ratio of the insertion chimeras to the pair-end chimeras. For this purpose, we firstly hypothesized that the chimeric probability happening on each nucleotide of the PE reads was equally distributed. Since the sequenced length of the PE reads were constant (202 nt), theoretically the average insertion size was the principal parameter. The sequence data of the sample SRX247249 was divided into 18 parts according to the run number. Then their average insertion sizes and the ratios of the insertion chimeras to the pair-end chimeras were calculated (Table A in S1 file). The results revealed the significant positive correlation (R^2^ = 0.9691) between the two indexes (Fig 5).

## Discussion

In this study, we finished a further analysis towards the phi29MDA human genome sequence data of the whole genome haplotype assembling. We established a bioinformatics pipeline to comprehensively illustrate several aspects of the phi29MDA chimeras, *e*.*g*. the visualization of typical chimeras, the level classification of all varied chimeras, the proportion statistics of the varied-level chimeras, and the distribution statistics of two artificially defined indexes. The statistically significant total proportion of chimeras in WGA sequence data illustrated the importance to systematically study this special type of sequence data, which was easily ignored in genomic researches based on phi29MDA.

Normally, according to the different parameter settings in reads alignments, insertion chimeras could be regarded as wasted data in many researches using *de novo* sequencing, because they were classified as the single-end (SE) mapped reads in the standard SOAP2 alignment results [17]. Meanwhile, the pair-end chimeras were undoubtedly abandoned as wasted data in almost all researches using NGS. However, these chimeras could be translated into multiple-fragments available data through our analytical pipeline. Therefore, our study contributed to the improving of the sequence data utilization efficiency.

During the process of phi29 MDA, strand displacements are happening with a high frequency, which leads to the construction of the hyperbranched structure. Herein, due to the ultrahigh complexity and scale of the human genome, almost all DNA polymers are always “facing” countless binding positions and would bind to one of them which could keep the polymers in most energetically stable condition. According to this inference, DNA polymers definitely tend to choose one close position with relatively long overlap segments generally. Therefore, inverted chimeras are easier to be generated according to its relatively simpler structure; on the contrary, direct chimeras are more difficult to be generated thermodynamically because of the complex structure. On the same principle, low-level chimeras are easier to be generated (Figure C in S1 file).

Since the insertion chimeras are directly available while the pair-end chimeras could be easily classified as unmapped reads, it is necessary to discovery the influencing factor towards the ratio between the two categories of chimeras. We used data of the sample SRX247249 to construct 18 subsamples according to the run numbers, in order to see the relationship between the average insertion sizes and the ratios between the two categories of chimeras, and found the significant positive correlation between two indexes. The results revealed that proper increasement of the average insertion size in standard DNA library construction for Illumina Sequence Platform was effective for the proportion reduction of the pair-end chimeras. As for the aspect of bioinformatics analysis, after being cut into two or more subsections according to the chimeric sites through our pipeline, the segments of chimeras could be integrated into a new FASTQ file, which would be aligned to the reference genome again. Thus, the chimeras are recycled instead of being thrown into the unmapped wasted data. By comprehensively using both experimental and bioinformatics solutions, researchers are able to increase the sequence data utilization efficiency with this strategy.

In comparison with previous researches about phi29MDA chimeras, reanalyzed data in our study was sequenced from human genome, which is more complex structurally and dimensionally than genomes of prokaryotes. Due to the extreme complicacy of the human genome, the generation of a great quantity of chimeras in DNA isothermal amplification may not only reflect the genomic revolution from simple prokaryotes to complicated Homo Sapiens, but also be persuasive to explain the general sequence symmetry of human genome, *i*.*e*. the Chargaff 2^nd^ Parity Laws [18].

In spite of the chimera generation, phi29 DNA polymerase is undoubtedly worth being used in many WGA experiments based on its obvious advantages. Our study broadens the horizons about the enzyme kinetics of the phi29 DNA polymerase, and may be helpful for the comprehensive explanation of the thermodynamics of the phi29MDA process. Furthermore, the unique multiple-fragments structure of the chimeras illustrates the potential value in future studies of human genome [19].

## Materials & Methods

### Data Sources

Genome sequence data was downloaded from National Center for Biotechnology Information (NCBI) Sequence Read Archive (SRA) database, and the SRA numbers were SRX247249 and SRX252522. The type of the sequence data was PE–101.

### Bioinformatics pipeline establishment

The phi29MDA chimeras could be divided into two categories: the insertion chimeras and the pair-end chimeras. Since we could find the insertion chimeras by directly observing the mapping locations (+ or—strands) of the PE reads and judging their logical relation, we established a bioinformatics pipeline based on the subsection alignment strategy for the purpose of the detailed analysis to the pair-end chimeras. The pipeline could be mainly divided into two parts: A. reads alignment and filtering; B. detailed analysis of the candidate chimeras.

A. Reads alignment and filtering. Firstly we obtained the PE–101 clean reads after removal of the PE–101 raw reads with N. The clean reads would be conducted in two ways. On one hand, they were mapped to the Hg19 human genome reference by using SOAP2 software in PE mode. From the alignment output data, we abandoned the PE mapped reads, and kept the other reads (including SE mapped reads and unmapped reads) for the following analysis. On the other hand, we cut 30-nt seeds from the clean data, and constructed a series of PE–30 clean reads. These reads were also mapped to the Hg19 human genome reference by using SOAP2 software in PE mode. From the alignment output data, we abandoned the unmapped reads, and kept the other reads (including PE and SE mapped reads) for the following analysis. According to the reads ID, we found the PE–101 reads from the clean data with the characteristics described as: their 30-nt seeds could accurately mapped to the genome, while they could not on the same location. These reads would be collected as the candidate reads for the following detailed analysis about the pair-end chimeras (Figure A in S1 file).

B. Subsection alignment strategy used for chimera search. In this step, we aimed to accomplish the discovery of all the pair-end chimeras, meanwhile understand their exact structure. This step mainly included two core substeps: seed extension and local alignment. Initially, the mapped 30-nt seeds were extended one nucleotide by one nucleotide on their related whole reads until they arrived at a mismatch site. Here we obtained the two subsections of the pair-end chimeras and the start-end coordinates of the former subsections on the reference genome. Afterwards, the following subsections of the pair-end chimeras were locally aligned in the 5,000 bp local region both upstream and downstream from the end coordinate of the former subsections in order to find their exact location in the genome. Thus we could know start-end coordinates of the following subsections of the pair-end chimeras. Finally, we reversely extended the following subsections one nucleotide by one nucleotide from their start coordinates on the reference genome, and compared the nucleotide type with the end of the former subsections, in order to obtain the overlap segments (Figure B and Figure D in S1 file).

According to the start-end coordinates of the two subsections of the pair-end chimeras, we could calculate the chimeric distance through the equation:

$$D=C_{former,\;end}–C_{following,\;start}.$$

In the equation, *D* meant the chimeric distances, *C*_*former*, *end*_ meant the end coordinates of the former subsections, and *C*_*following*, *start*_ meant the start coordinates of the following subsections.

## Supporting Information

S1 File Supporting figures and tables. (PDF)

## Abbreviations

MDA: multiple displacement amplification
WGA: whole genome amplification
NGS: next generation sequencing
SOAP2: Short Oligonucleotide Alignment Program 2
DOP-PCR: degenerate oligonucleotide primed polymerase chain reaction
PE: pair end
SE: single end
nt: nucleotide
bp: base pair
