# Supplementary material for: Systematic Characteristic Exploration of the Chimeras Generated in Multiple Displacement Amplification through Next Generation Sequencing Data Reanalysis
Source: PLoS One. 2015 Oct 6;10(10):e0139857. doi: 10.1371/journal.pone.0139857 (PMC4595205; doi:10.1371/journal.pone.0139857)
Supplement: S1 File — (PDF) [file pone.0139857.s001.pdf]

Supporting information

Figures

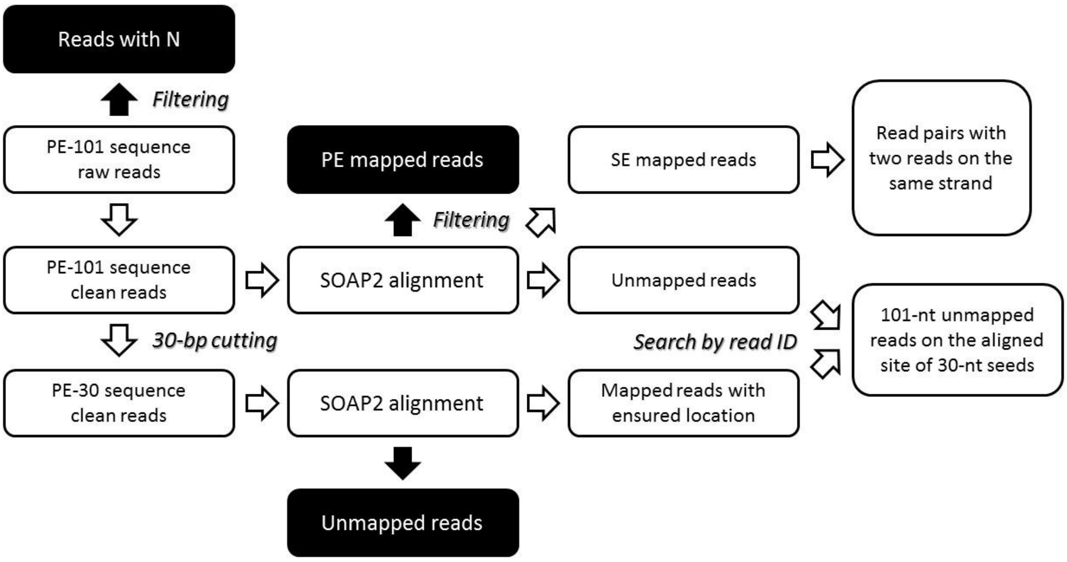

Figure A: Reads alignment and filtering

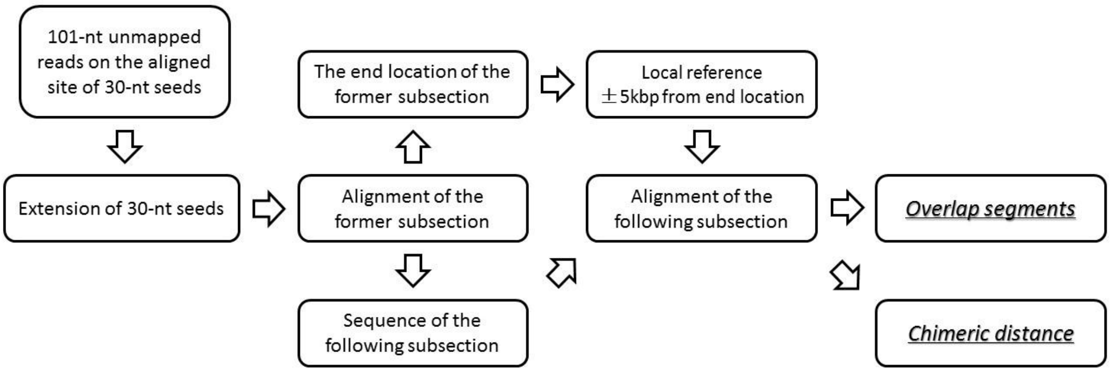

Figure B: Subsection alignment strategy used for the chimera discovery

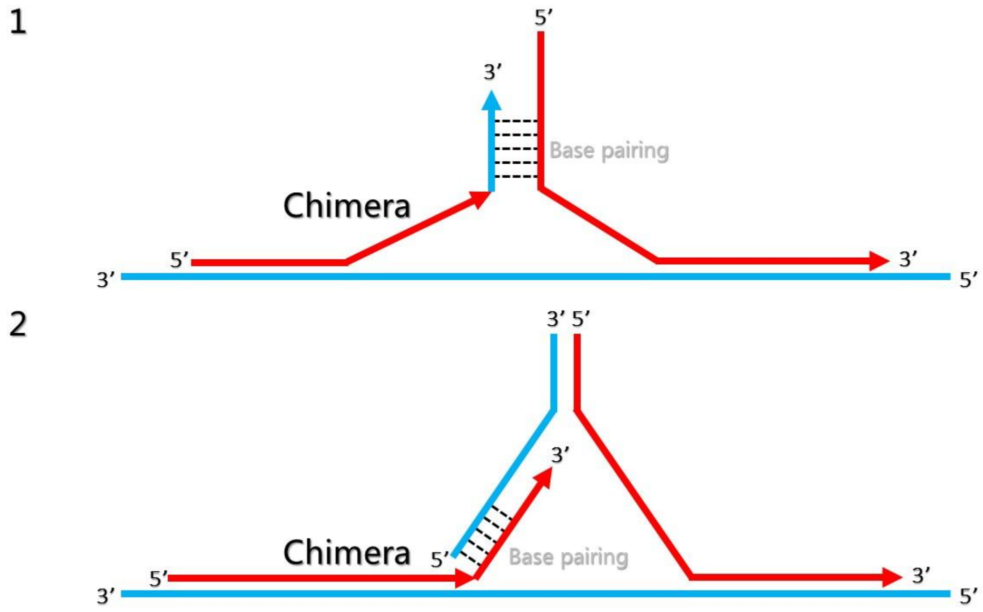

Figure C: The probable generating process of phi29 MDA chimeras based on the hyperbranched structure.

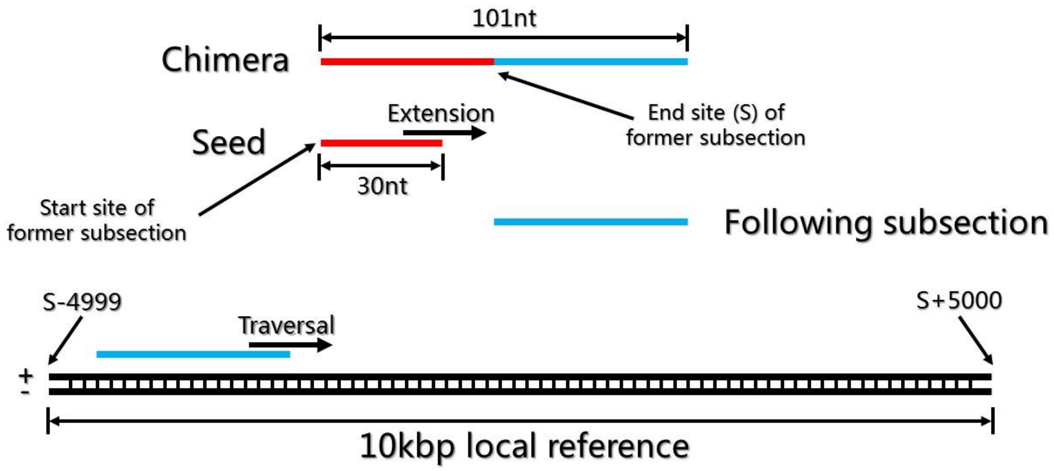

Figure D: Demonstration of the two core substeps (**seed extension** and **local alignment**) in the subsection alignment strategy.

Tables

**Run number**	**Read pairs**	**Average insertion size**	**Insertion chimera**	**End chimera**	**Chimera ratio***
**SRR799550**	13371952	211.78	663962	213881	3.104
**SRR799549**	17068542	212.73	837201	269295	3.109
**SRR799560**	14184567	233.13	786364	232254	3.386
**SRR799545**	18297984	234.36	1021312	301806	3.384
**SRR799547**	14620324	237.41	1151644	337438	3.413
**SRR799561**	14456680	239.76	1106215	319198	3.466
**SRR799558**	17646526	240.47	1397000	404868	3.451
**SRR799552**	13365660	241.40	1108055	319539	3.468
**SRR799546**	15580764	241.72	917696	263291	3.485
**SRR799553**	16158737	242.00	945228	273189	3.460
**SRR799559**	18842904	242.45	1077455	311421	3.460
**SRR799548**	16156682	243.19	1337308	389258	3.436
**SRR799556**	16836834	243.31	973698	282962	3.441
**SRR799557**	15275461	243.52	1268411	365002	3.475
**SRR799551**	14445390	244.13	1234888	356872	3.460
**SRR799544**	14641175	244.46	870838	251165	3.467
**SRR799554**	13765673	244.70	1192499	342578	3.481
**SRR799555**	10802212	247.27	927978	264538	3.508

Table A: The data illustration of the 18 subsamples about the relationship between the average insertion size and the ratio of the insertion chimeras to the end chimeras

*Chimera ratio = Insertion chimeras / End chimeras
